# Synthesis and Evaluation of Melittin-Modified Peptides for Antibacterial Activity

**DOI:** 10.3390/toxins17020098

**Published:** 2025-02-19

**Authors:** Xiangxiang Xu, Hongyi Fu, Weihui Wu, Liang Zong, Dan Li, Bo Zhuang, Yelin Qi, Xiuli Qi, Ting Liang

**Affiliations:** Institute of NBC Deffence, Beijing 102205, China; 17710315316@163.com (X.X.); 18729592385@163.com (H.F.); wuwh2012@163.com (W.W.); 17718562802@163.com (L.Z.); 13389705203@163.com (D.L.); zhuangbo007@163.com (B.Z.); fhqiyelin@163.com (Y.Q.)

**Keywords:** melittin-modified peptide, solid-phase synthesis, antimicrobial property

## Abstract

Melittin, a naturally occurring antimicrobial peptide, demonstrates broad-spectrum activity, effectively suppressing and eliminating both Gram-positive and Gram-negative bacteria, including specific drug-resistant strains. In this study, molecular simulation software was employed to investigate and modify the structure of melittin with the aim of synthesizing a modified peptide exhibiting enhanced antibacterial potency and assessing its bacteriostatic and antibacterial properties. The primary research objectives were as follows: 1. Preparation and characterization of melittin-modified peptide—Using molecular simulation software, the structure of the melittin-modified peptide was adjusted to predict its activity and select the most appropriate amino acid sequence. The peptide was synthesized through solid-phase peptide synthesis employing the Fmoc strategy and subsequently purified using liquid chromatography. The yield of the purified modified melittin was determined to be 30.97%, and the identity of the product was confirmed by LC-MS and MALDI-TOF-MS. 2. Evaluation of the antimicrobial activity of the melittin-modified peptide—The minimum inhibitory concentration (MIC) and minimum bactericidal concentration (MBC) of melittin and its modified peptide were measured using gradient dilution and plate counting techniques. The results revealed that both melittin and its modified peptide exhibited strong antibacterial efficacy against Gram-positive and Gram-negative bacteria, as well as certain drug-resistant strains. This showed that melittin and its modified peptide have the same antibacterial (killing) effect. A scanning electron microscope analysis indicated that both melittin and its modified peptide were capable of disrupting bacterial cell structures, leading to bacterial cell death.

## 1. Introduction

The persistent increase in bacterial mutation rates, exacerbated by the overuse of antibiotics, has intensified the antibiotic resistance crisis, rendering it a significant global concern. Antibacterial peptides are distinguished by their broad-spectrum antibacterial activity for bacteria, viruses, and tumor cells. Their potent inhibitory effects have attracted considerable attention within the scientific community [1].

Natural antibacterial peptides display significant diversity and are sourced from various origins, with insect-derived peptides forming a prominent subgroup. Melittin, extracted from bee venom, exemplifies a class of venom-derived peptides. This cationic linear amide peptide consists of 26 amino acids and exhibits amphipathicity in its primary sequence. At a physiological pH, melittin carries a net charge of +6 [2]. Its secondary structure predominantly assumes a canonical amphipathic α-helical conformation [3]. The presence of proline residues within this α-helical framework causes the peptide backbone to kink at a specific angle [4]. Research has clearly shown that upon interaction with biological membranes, melittin’s secondary structure folds at a defined angle, which is believed to aid in the disruption of bacterial cell membranes, thereby augmenting melittin’s antibacterial efficacy [5]. Melittin inserts into the cell membrane in a directional manner, forming cavities on the bacterial membrane surface. Upon penetration, melittin can hydrolyze DNA and RNA, and it exerts an inhibitory effect on protein synthesis. A study by Fennell in 1968 demonstrated that melittin exhibits antibacterial activity against penicillin-resistant strains of *Staphylococcus aureus* [6]. In 2002, Lazarev et al. confirmed that melittin can impair human *Mycoplasma* and *Chlamydia* trachomatis infections through a cytotoxic mechanism [7]. Research by Pan in 2008 revealed that melittin exhibits significant inhibitory effects on Gram-negative and Gram-positive bacteria, fungi, and certain mold spores [8].

In recent years, melittin has attracted significant attention in the emerging field of combination therapies with traditional antibacterial agents, such as antibiotics. This has been supported by previous research. Guha et al. [9] provided an overview of the rich history of scientific research into the many activities of melittin, highlighting its potential in antibacterial applications. Kmeck et al. [10] also discussed the synergies and resistance related to membrane-active peptides, including melittin. In 2022, Rasoul Mirzaei et al. [11] demonstrated the efficacy of melittin against multi-drug-resistant *Methicillin-Resistant Staphylococcus aureus* (MDR-MRSA) isolates. The therapeutic indices of the melittin–vancomycin and melittin–rifampicin combinations were found to be 32.08-fold and 12.82-fold higher, respectively, than those of the antibiotics when used individually, indicating a notable degree of synergy and representing a promising strategy for the treatment of *MDR-MRSA* infections. Studies show that melittin has anti-tumor effects. It can inhibit tumor cell growth, metastasis, migration, and invasion, reduce cell viability, and induce apoptosis. In 2020, research indicated its therapeutic effect on HPV-positive cervical cancer cells [12]. Also in 2020, it was found to inhibit bladder cancer cell growth [13]. In 2022, it was shown to regulate breast cancer cell metastasis and viability [14]. In 2023, it was reported to inhibit non-small cell lung cancer cell proliferation [15]. This study aimed to utilize bioinformatics methodologies and molecular simulation software to investigate the structure of melittin, analyze factors influencing its antibacterial efficacy, and modify and synthesize melittin. Additionally, we conducted antibacterial efficacy assays against various bacterial strains. The objective was to develop a modified melittin peptide that exhibits enhanced antibacterial performance, thereby providing a valuable framework for the structural modification of melittin to combat drug-resistant bacterial strains.

## 2. Results

### 2.1. Structural Modification and Activity Prediction of Melittin

**Figure 1:**
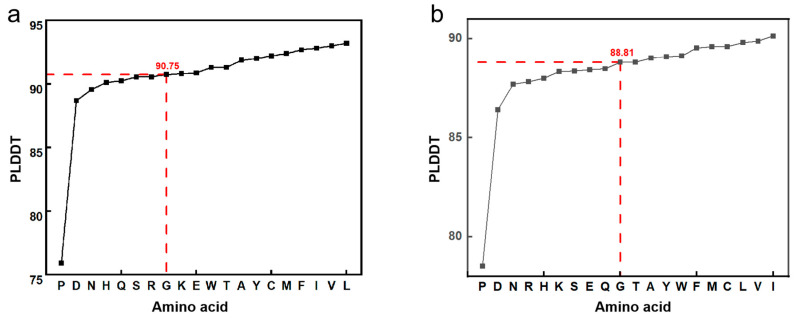
The trend chart of the PLDDT of the melittin-modified peptide. (**a**) The trend diagram of the variation in PLDDT at a single site of the melittin-modified peptide among the 20 samples. (**b**) The trend diagram of the variation in PLDDT across the sequences of the 20 melittin-modified peptides.

**Figure 2:**
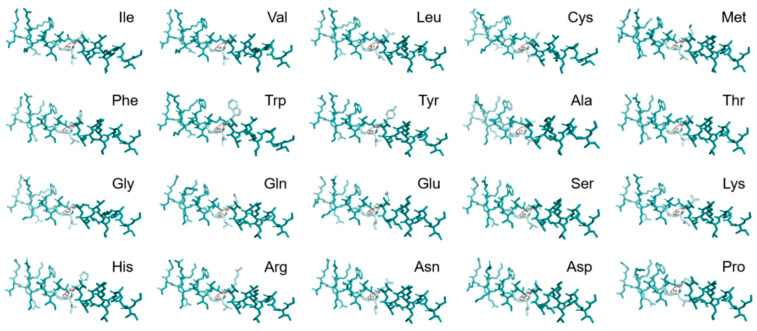
Schematic representation of the angle between the central carbon atoms at different positions.

The PLDDT was employed as the metric for evaluating the confidence level of the melittin-modified peptide. Residues with PLDDT values exceeding 90 were classified as exhibiting high confidence. Residues with PLDDT values ranging from 70 to 90 were categorized as having medium to high confidence. Conversely, residues with PLDDT values below 50 were considered potentially disordered structures or isolated non-structural elements. The variations in PLDDT at individual sites and throughout the peptide sequences of 20 modified melittin peptides were plotted, as depicted in Figure 1. This study employed PyMOL software to visualize multiple sequences of melittin-modified peptides and construct a fundamental model. The angle that was formed by the central carbon atoms of the 12th to 14th amino acids in the melittin backbone indicated the angle between the N-terminal α-helix and the C-terminal α-helix of the melittin-modified peptides, as illustrated in Figure 2. The predicted data for the angles between the α-helices at both termini of the 20 arranged melittin-modified peptides are presented in Table 1, providing a comprehensive and quantitative summary of the structural characteristics that were under investigation. In the experiment, Ala was selected to replace the 12th Gly of melittin for the purpose of realizing the structural transformation of melittin. Due to its simple hydrophobic side chain structure, Ala is capable of increasing the α helix angle between the two ends of melittin, thereby enhancing the binding ability of melittin to the bacterial cell membrane while not altering the charge number of melittin. It has no significant influence on the folding of the secondary structure of the peptide chain.

### 2.2. Melittin-Modified Peptide Characterization

**Figure 3:**
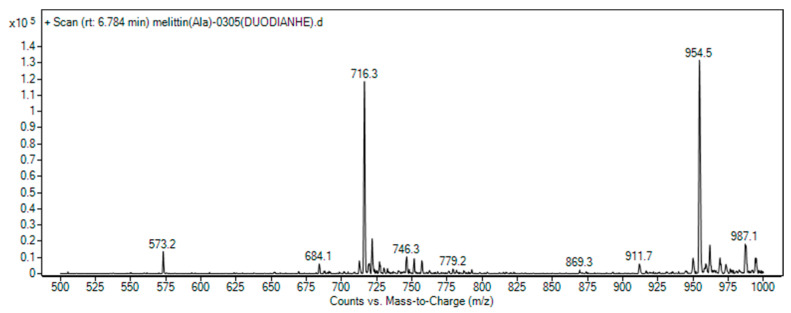
LC-MS spectrum of melittin-modified peptide.

**Figure 4:**
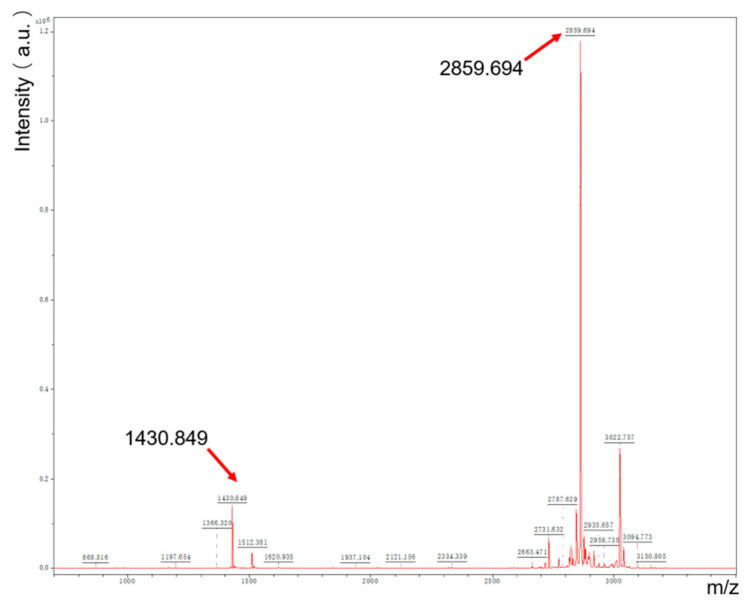
MALDI-TOF-MS of melittin-modified peptide.

**Figure 5:**
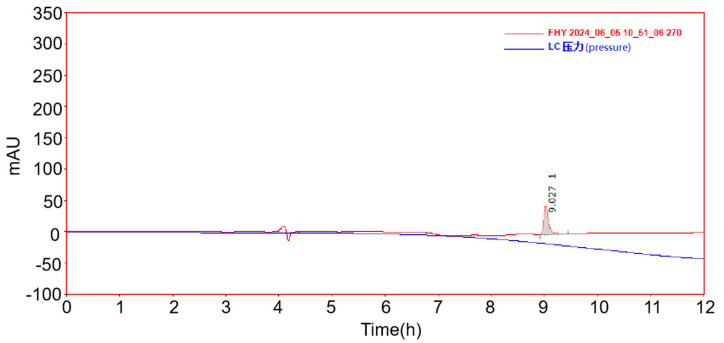
Liquid chromatogram of melittin-modified peptide.

The characterization of the melittin-modified peptide was conducted using the LC-MS and MALDI-TOF-MS techniques. In the LC-MS analysis, the observed mass-to-charge ratios (*m*/*z*) of 716.3 and 954.5 corresponded to the ions [M + 4H]^4^⁺/4 and [M + 3H]^3^⁺/3, respectively. The theoretical values for these ions were 715.9 and 954.3, thereby confirming the purified sample as the melittin-modified peptide (see Figure 3). Supporting data from MALDI-TOF-MS indicated the molecular weight of the product, with peaks at 2859.694 and 1430.849 being observed (see Figure 4). The former represents the molecular ion peak, while the latter corresponds to a doubly charged ion peak associated with [M + 1]⁺ and [M + 2]^2^⁺/2. The theoretically calculated molecular weights of these ions were 2960.7 and 1430.9, respectively, confirming the peptide’s identity. After a vacuum-drying process lasting four hours, the Rink-amide PEG matrix resin demonstrated an increase in weight of 0.202 g, indicating successful conjugation with the melittin-modified peptide chain, which retained side chain protection. The relative molecular weight of the protected melittin was found to be 4401.62. The crude yield of melittin was 91.78%, and subsequent processing and lyophilization of the crude peptide resulted in a yield of 44.27 mg. A total of 0.05 mmol of the peptide was synthesized using Liberty Blue, with a theoretical yield of 142.94 mg and an actual pure product yield of 30.97%. The purity of the peptide was elucidated using HPLC (see Figure 5). A peak observed prior to 9.027 min in the chromatogram was attributed to mobile phase switching and peptide solubility, rather than an impurity. The peptide peak was distinctly registered at 9.027 min, with no additional impurity peak being detected, confirming a purity of 100%.

### 2.3. Antibacterial Performance Outcome

#### 2.3.1. Comparison of Antibacterial Rate Between Melittin and Melittin-Modified Peptide

**Figure 6:**
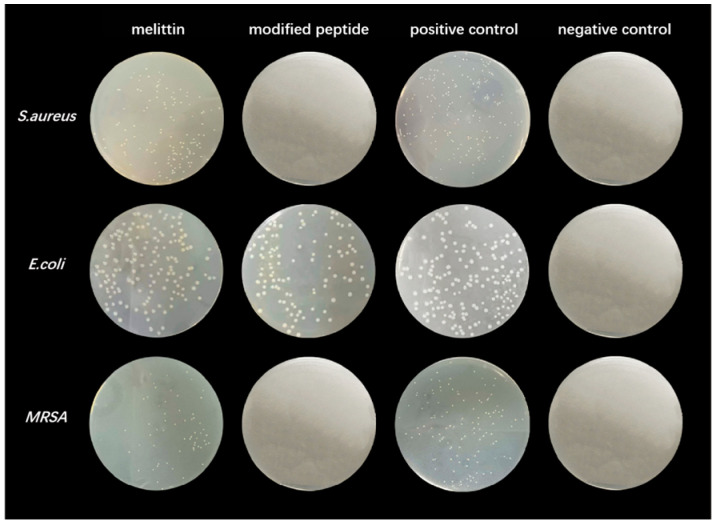
Plate maps of three bacteria treated with melittin and its modified peptide at 2 μg/mL.

The average bactericidal rates of *S. aureus*, *E. coli*, and methicillin-resistant *MRSA* for various concentrations of melittin and its modified peptide were calculated (refer to Table 2 and Table 3). An analysis of the data from these tables was conducted alongside the assessment of colony growth in bacteria that were treated with 2 μg/mL of both melittin and its modified peptide (see Figure 6). At a bacterial concentration of 2 × 10^2^ colony-forming units per milliliter (CFU/mL), both 4 μg/mL melittin and 2 μg/mL of the modified peptide exhibited 100% antimicrobial efficacy against *S. aureus* and *MRSA*. For *E. coli*, melittin and the modified peptide displayed similar antibacterial trends, with the modified peptide demonstrating slightly higher effectiveness. The antibacterial activity against *E. coli* was less pronounced than that against *S. aureus*, likely due to differences in cell structure or binding sites. Additionally, 2 μg/mL melittin achieved a higher antimicrobial rate against *MRSA* compared to *S. aureus*. The comparative analysis of the percent inhibition indicated that the modified peptide exhibited superior efficacy compared to melittin against these bacteria. The increase in the α-helix angle at both termini of melittin enhanced the local hydrophobicity of the peptide chain, improved the stability of the secondary structure, and augmented its antibacterial and bactericidal properties. These findings provide insights into the structural modifications of melittin and their effects on antibacterial activity, which may inform the development of more effective antimicrobial agents.

#### 2.3.2. MIC and MBC of Melittin and Melittin-Modified Peptide

*S. aureus* was selected as the representative Gram-positive bacterium, *E. coli* for Gram-negative, and *MRSA* for drug-resistant bacteria. At a concentration of 3.2 μg/mL or lower, solutions of *S. aureus*, *E. coli*, and *MRSA* exhibited turbidity after 24 h of incubation. However, at concentrations of 6.4 μg/mL or higher, these solutions became clear, exhibiting optical density (OD600) values that were comparable to those of the negative control. The MIC of melittin for these bacteria was determined to be 6.4 μg/mL, based on an initial bacterial concentration of 5 × 10^5^ CFU/mL. For the melittin-modified peptide, the same turbidity was observed at concentrations of 3.2 μg/mL or lower after 24 h. At 6.4 μg/mL or higher, the solutions were likewise clear, showing OD600 values that were similar to the negative control, with the MIC also at 6.4 μg/mL for the same initial concentration. These results provide insights into the antibacterial activities, highlighting the importance of MIC determination. The similar MICs suggest that the modification of melittin did not significantly alter the inhibitory concentration. Future studies could investigate the differences in mechanisms and killing kinetics. The experimental data indicate that treatment with melittin and its modified peptide at concentrations of 6.4 μg/mL or higher effectively eliminated turbidity in solutions of *S. aureus*, *E. coli*, and *MRSA* after 24 h of incubation. Observations of the bacterial colony growth on agar plates that were treated with melittin at concentrations of 51.2, 25.6, 12.8, and 6.4 μg/mL revealed no colony formation for *S. aureus*, *E. coli*, and *MRSA*. Consequently, for an initial bacterial concentration of 5 × 10^5^ CFU/mL, the MBC of melittin and its modified peptide against *S. aureus*, *E. coli*, and *MRSA* was found to be 6.4 μg/mL. Moreover, results from both the MIC and MBC experiments demonstrated that at a bacterial concentration of 2 × 10^5^ CFU/mL, both the MIC and MBC of melittin and its modified peptide for *S. aureus*, *E. coli*, and *MRSA* remained consistently at 6.4 μg/mL.

#### 2.3.3. Bacterial Growth Curve of Melittin and Its Modified Peptide

**Figure 7:**
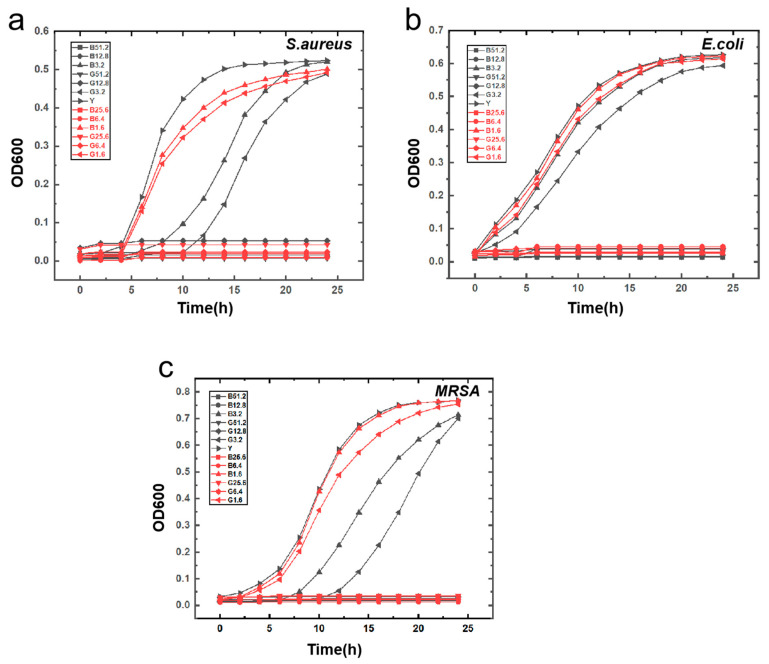
The bacterial growth curves of the three bacteria in response to the action of melittin and its modified peptide. (**a**) The growth curve of *S. aureus* bacteria under the influence of melittin and its modified peptide; (**b**) the growth curve of *E. coli* under the impact of melittin and its modified peptide; (**c**) the growth curve of *MRSA* under the effect of melittin and its modified peptide.

**Figure 8:**
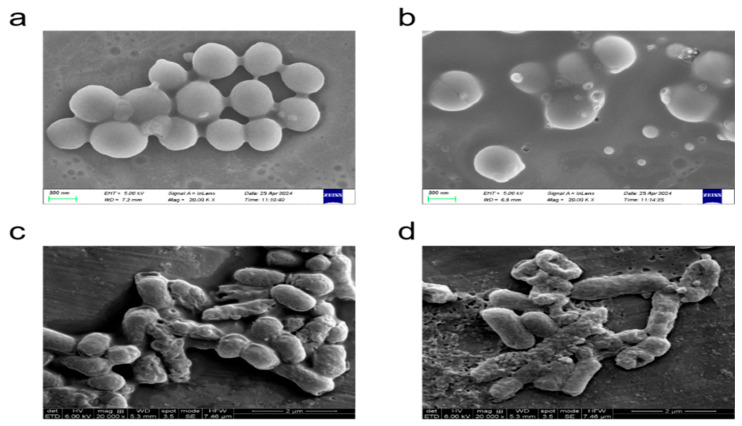
The effect of melittin and its modified peptides on different genus of bacteria. (**a**) The morphological structure of *S. aureus* upon treatment with melittin, (**b**) the morphological structure of *S. aureus* following treatment with the melittin-modified peptide, (**c**) the morphological structure of *E. coli* after treatment with melittin, and (**d**) the morphological structure of *E. coli* following melittin-modified peptide treatment.

The bacterial growth curves of *S. aureus*, *E. coli*, and *MRSA* induced by melittin and its modified peptide were constructed. The absorbance at 600 nm was measured for each, and the bacterial growth curves of *S. aureus*, *E. coli*, and *MRSA* within 24 h were plotted (Figure 7). By analyzing the growth trend of the *S. aureus* growth curve in Figure 8, it was observed that melittin and its modified peptide at a concentration of 6.4 μg/mL or higher could effectively inhibit the growth of *S. aureus*. When comparing the change trends of the bacterial growth curves of 3.2 μg/mL and 1.6 μg/mL melittin and its modified peptide, it was evident that the antibacterial activity of the melittin-modified peptide at 3.2 μg/mL and 1.6 μg/mL was superior to that of melittin at the same concentrations. The experimental results demonstrated that the antimicrobial and antibacterial activities of the modified peptide were enhanced as a result of the structural modification of melittin compared to the unmodified melittin.

#### 2.3.4. Scanning Electron Microscope

The influence of melittin and its modified peptide on the morphological architecture of *S. aereus* and *E. coli* was meticulously examined using scanning electron microscopy (Figure 8). Upon exposure to melittin and its modified peptide, the bacteria underwent significant alterations, characterized by the roughening of their surfaces, which indicates initial impacts on the integrity of the cell envelope. The structural components that are critical for maintaining normal physiological functions and bacterial viability were severely compromised. This damage manifested as disruptions and deformations, resulting in pronounced distortions of the cell morphology. Notably, some cells exhibited adherence to one another, a phenomenon that is potentially attributable to alterations in their surface properties and the release of intracellular components, leading to aggregate formation. The findings from the electron microscopy unequivocally demonstrated that melittin and its modified peptide can disrupt and dismantle bacterial cell structures. By impairing the structural integrity, these peptides effectively interfere with fundamental processes that are essential for bacterial survival, including nutrient and metabolite transport across the cell membrane, the maintenance of osmotic balance, and proper functioning of the genetic machinery. Consequently, this disruption facilitates efficient bacterial killing, underscoring the potential of melittin and its modified peptide as potent antibacterial agents. The detailed visualization of these morphological changes not only vividly demonstrates direct antibacterial effects but also provides insights into the underlying mechanisms of action, allowing for a more profound understanding of how melittin and its modified form interact with bacterial cells at a structural level. Such knowledge is essential for unraveling the complex processes that are involved in antibacterial activity. Furthermore, comparing the morphological alterations that are induced by melittin and its modified peptide may reveal differences in their modes of action, which could be leveraged for optimizing and developing more effective antibacterial strategies.

## 3. Discussion

### 3.1. Structural Modification and Activity Prediction of Melittin

**Figure 9:**
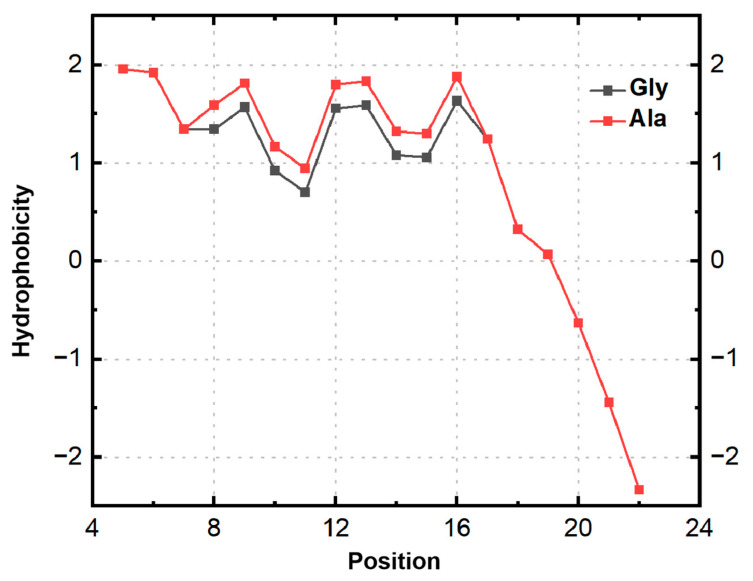
Predicted alterations in the hydrophobicity of melittin and its modified peptides at different positions.

Considering the helix–hinge–helix structure and its mechanism for penetrating cell membranes, the Gly-X-Pro sequence in the hinge region serves as a prevalent turning motif. Melittin experiences torsion and kinking within the Gly-X-Pro hinge region, generating a spatial angle between the N-terminal α-helix and the C-terminal α-helix. The presence of this hinge region facilitates the folding of melittin’s secondary structure, which influences the binding affinity of the α-helices at both termini to the phospholipid bilayer of the bacterial cell membrane. Notably, proline residues, due to their five-part ring structure, resist rotation and lack amide hydrogen bonds, rendering them incapable of forming intra-chain hydrogen bonds. Consequently, proline residues play a key role in determining the rotation angle of melittin’s main chain. Accordingly, in this study, we selected the 12th amino acid of melittin as the modification site to achieve structural modifications of the original melittin sequence through the prediction of activities that are associated with various modified peptide sequences. The confidence levels of single-site and local regions of 20 melittin-modified peptides were predicted. The PLDDT values for single sites and the entire peptides were 90.75 and 88.81, respectively. Substituting specific amino acids for the 12th Gly of melittin resulted in higher confidence levels for the modified peptide at both single sites and local regions, indicating a more stable secondary structure. An analysis of the predicted α-helix angles revealed that some amino acid substitutions increased the angles between the central carbon atoms at positions 12–14 of melittin, while others decreased them. Overall, the analysis indicated that increased α-helix angles at both ends of the melittin-modified peptide enhanced peptide conservation and the secondary structure’s stability. Based on the binding mechanism, replacing the 12th Gly in melittin alters the α-helix angle and folding angle in the hinge region, thereby enhancing the binding affinity with the bacterial cell membrane and optimizing the antibacterial performance. In the experimental procedure, alanine (Ala) was selected to replace glycine (Gly) at the 12th position of melittin, resulting in a structural alteration of the peptide. Due to its straightforward hydrophobic side chain architecture, Ala can increase the α-helix angles at both termini of melittin, thereby enhancing its binding affinity to the bacterial cell membrane while preserving the overall charge of melittin. Additionally, because of its simple side chain structure, Ala exerts minimal impact on the folding conformation of the secondary structure of the peptide chain. The hydrophobicity predictions for each amino acid site of melittin and its modified peptide, as determined by Empasy (Figure 9), further indicate that the modified peptide exhibits greater average hydrophobicity for the amino acids located at positions 8–16 compared to melittin.

### 3.2. Modification of Melittin Structure and Synthesis of Modified Peptides

In the transformation of melittin, molecular simulation software—including Discovery Studio, the Alphafold2 protein 3D structure prediction model, and PyMOL visualization software—was utilized to simulate the structures of melittin and several modified peptides. Various influencing factors were analyzed, including the PLDDT, α-helix angles at both ends of the modified peptide, peptide chain conservation, secondary structure stability, hydrophobicity, and the number of charges. It was observed that the PLDDT increased, the α-helix angles rose at both ends of the modified peptide, the peptide chain conservation and secondary structure stability improved, and the hydrophobicity increased.

During the synthesis and preparation of the modified peptides, the Fmoc strategy was employed. The yield of the modified melittin peptide was determined to be 30.97%, with a purity of 100%, as established by the area normalization method. In the solid-phase synthesis, due to the challenge of coupling Fmoc Arg, arginine was conjugated twice, and the microwave temperature was maintained at 75 °C to improve the coupling yield and minimize the formation of defective peptides. Ultimately, the purity of the melittin-modified peptide was determined to be 71.8%. The crude modified peptide was isolated and purified using preparative liquid chromatography, and successful purification was achieved by adjusting the minimum collection threshold of the sample discharge peak multiple times.

### 3.3. Evaluation of Antibacterial Effect

When the bacterial concentration was 2 × 10^4^ CFU/mL, the antibacterial efficacy of 4 μg/mL melittin and 2 μg/mL modified peptide against *S. aureus* and *MRSA* reached 100%, indicating that the antibacterial capacity of the modified peptide was at least doubled relative to the original peptide. The next step involved identifying the inflection point in the antibacterial rate based on 2 μg/mL of the modified peptide. The antibacterial trends of melittin and its modified peptide against *E. coli* remained consistent, with the modified peptide demonstrating a higher antibacterial rate than melittin to a certain extent. The antibacterial activity of both melittin and its modified peptides against *E. coli* was comparatively weaker when contrasted with *S. aureus*, potentially due to differences in the cell structure or binding sites. Notably, at a concentration of 2 μg/mL, the antibacterial rate of melittin against *MRSA* was greater than that against *S. aureus*, an observation warranting further investigation. The antibacterial rate data indicate that the melittin-modified peptide exhibits improved efficacy against *S. aureus*, *E. coli*, and *MRSA* compared to melittin alone. This finding validates our original molecular design hypothesis, specifically that increasing the angle between the α helix at both ends of melittin, enhancing the local hydrophobicity of the peptide chain, and improving the stability of the melittin’s secondary structure positively influence its antibacterial ability and bactericidal efficacy. At a bacterial concentration of 2 × 10^5^ CFU/mL, the minimum inhibitory concentration and minimum bactericidal concentration of melittin and its modified peptide for *S. aureus*, *E. coli*, and *MRSA* were both 6.4 μg/mL, signifying that antimicrobial peptides can efficiently eliminate microorganisms while inhibiting their growth. According to the bacterial growth curve, the melittin-modified peptide demonstrated enhanced antibacterial characteristics and efficacy compared to melittin. The scanning electron microscopy results illustrated that both melittin and its modified peptide disrupt the bacterial cell structure effectively, leading to bacterial cell death.

## 4. Conclusions

In this study, molecular simulation techniques were employed to modify the structure of melittin and predict its activity, thus achieving the synthesis and preparation of melittin-modified peptide antibacterial materials and exploring the antibacterial properties of the modified peptide. When determining the amino acid sequence of the melittin-modified peptide, based on the factors influencing the structure and antibacterial performance of melittin, and by virtue of the comprehensive and efficient features of bioinformatics, molecular simulation software was innovatively used for structural modification and activity prediction of melittin, and PyMOL was used for visualizing the melittin-modified peptide. The amino acid sequence of the fitted melittin-modified peptide was screened and determined. The evaluation of antibacterial effects showed that the antibacterial performance of the modified peptide was improved to some extent compared with that of melittin, confirming our hypothesis.

This paper has reported the synthesis, preparation, and study of the antibacterial properties of a melittin-modified peptide, obtaining certain fundamental results. However, this research still has considerable room for improvement and research directions, which are worthy of further in-depth exploration. In future research, the optimal antibacterial concentration of the melittin-modified peptide against *S. aureus* and *MRSA* can be further explored, providing more favorable theoretical support for studying the antibacterial efficacy of the melittin-modified peptide against Gram-positive bacteria. The key amino acid sites in the hinge region of melittin can be modified to explore the influence of melittin’s structure on antibacterial performance and establish a new method for melittin structural modification based on bioinformatics, providing a clearer underlying method for finding antimicrobial peptides with good antibacterial performance. Melittin and its modified peptides can be conjugated with various antibiotics to break the bacterial resistance mechanism and achieve effective inhibition of multi-drug-resistant bacteria.

## 5. Materials and Methods

### 5.1. Materials and Instruments

Amino acid raw materials and Oxyma were sourced from Jill Biochemical (Shanghai, China) Co., Ltd. (Shanghai, China), N,N′-Methanetetraylbis(1-methylethylamine) (DIC) was obtained from Beijing Bo-maijie Technology Co., Ltd. (Beijing, China), and Trifluoroacetic Acid (TFA) originated from Shandong Xiya Chemical Co., Ltd. (Chengdu, China). Anhydrous diethyl ether, piperidine, and phenol were supplied by Sinopharm Chemical Co., Ltd. (Shanghai, China). Dichloromethane (DCM) was provided by Tianjin City Fuyu Fine Chemical Co., Ltd. (Tianjin, China), while piperidine and N,N-Dimethylformamide (DMF) were acquired from Beijing Chemical Factory (Beijing, China). LB broth and agar were sourced from Qingdao Haibo Biotechnology Co., Ltd. (Qingdao, China). *Staphylococcus aureus* ATCC 6538 (*S. aureus*), *Escherichia coli* ATCC25922 (*E. coli*), and *Methicillin-resistant Staphylococcus aureus* ATCC43300 (*MRSA*) were obtained from Beijing Biobest Biotechnology Co., Ltd. (Beijing, China). A UV–visible spectrophotometer was acquired from Shanghai Precision Instrument and Meter Co., Ltd. (Shanghai, China), and a Liberty Blue peptide synthesizer was sourced from CEM Corporation (Matthews, NC, USA). A dry nitrogen-blowing concentrator was provided by Shanghai Lichen Bangxi Instrument Technology Co., Ltd. (Shanghai, China), while the LC-MS system was obtained from Agilent Technologies Ltd. (Santa Clara, CA, USA). A preparatory liquid chromatograph was sourced from Waters Technologies (Shanghai) Co., Ltd. (Shanghai, China), and an analytical liquid chromatograph was supplied by Dalian Ylit Analytical Instrument Co., Ltd. (Dalian, China).

### 5.2. Design and Synthesis of Melittin-Modified Peptide

#### 5.2.1. Structural Modification and Activity Prediction for Melittin

Based on the helix–hinge–helix structure of melittin and its mechanism of penetrating cell membranes [5], Gly-X-Pro in the hinge region is a common turn sequence. Melittin undergoes rotational kinking at Gly-X-Pro in the hinge region, forming a spatial angle between the N-terminal α-helix and the C-terminal α-helix. Due to the presence of the hinge region, melittin folds in its secondary structure, which has a certain impact on the binding ability of its two-terminal α-helices to the phospholipid bilayer of the bacterial cell membrane. Among them, the Pro residue, with its five-part ring structure, is difficult to rotate, lacks an amide hydrogen, and cannot form intra-chain hydrogen bonds. It is the main amino acid that provides the rotation angle for the main chain of melittin. Therefore, in this paper, the 12th amino acid, Gly, of melittin was selected as the modification site. Through the activity prediction of different modified peptide sequences, the structural modification of the original melittin sequence was achieved. In this study, the AlphaFold2 protein three-dimensional structure prediction model (available at ColabFold) was utilized to substitute the 12th amino acid of melittin with each of the 20 natural amino acids. The amino acid sequence, formatted in FASTA, was input to generate the predicted structure of the modified peptide.

The parameter settings were as follows: num_queries: 20; use_templates: true; num_relax:0,relax_max_iterations: 200; ralax_tolerance: 2.39; relax_stiffness: 10.0; relax_max_outer_iterations: 3; msa_mode: mmseqs2_uniref_env; model_type: alphafold2_ptm; num_models: 5; and num_recycles: 3.

The Predicted Local Distance Difference Test (PLDDT) was selected as an indicator for evaluating the confidence level of the modified melittin peptide. PyMOL software 2.1 was employed to visualize multiple sequences of the modified melittin peptide to construct a fundamental model. This approach also facilitated the prediction of individual site confidence levels and the local region confidence within the modified melittin peptide.

#### 5.2.2. Synthesis of Melittin-Modified Peptide

The target compound was synthesized using the Fmoc solid-phase synthesis strategy. The amino acid sequence of the target peptide was identified as GIGAVLKVLTTALPALISWIKRKRQQ, with an amide moiety at the C-terminus. The primary solvent employed during the synthesis was DMF. DIC was used as the condensing agent, while Oxyma functioned as the activating agent. The deprotection reagent was a 20% solution of Piperidine in DMF. The selected resin was a Rink-amide PEG matrix resin with a specification of 0.18 mmol/g. The synthesis was conducted with a quantity of 0.05 mmol in high-swelling mode, and the resin swelling time was set for 900 s. In the coupling conditions, DMF served as the solvent, DIC as the condensing agent, and Oxyma as the activating agent. The quantities of the condensing agent and activating agent used were five times that of the resin. The standard coupling protocol involved coupling at 170 W and 75 °C for 15 s, followed by coupling at 30 W and 90 °C for 115 s. The cycle method was as follows: After the resin was conjugated with the first amino acid, it was subjected to Fmoc deprotection to expose the amino functionality. Subsequently, the exposed amino group was coupled with the carboxyl group of the second Fmoc-protected amino acid. After the coupling was complete, the Fmoc group on the second amino acid residue that was linked to the resin was removed, allowing the exposed amino group to be coupled with the carboxyl group of the third Fmoc-protected amino acid. This iterative process was repeated until the coupling and Fmoc protection of the last amino acid were completed. The resulting crude product was stored at a low temperature of 4 °C. After being washed with DMF and DCM, the peptide-loaded resin was dried under vacuum overnight, and then, the resin was subjected to cleavage. The cleavage solution was a TFA:H_2_O:phenol:TIPS system (88:5:5:2), and the cleavage time was 4 h. The melittin-modified peptide was isolated and purified using HPLC to obtain a purified product for further use. LC-MS and MALDI-TOF-MS were employed to characterize the pure melittin-modified peptide, providing comprehensive insights into its molecular structure and properties. The HPLC conditions are described below.

Column: We utilized an XBridge Peptide BEH C18 liquid chromatography column. This column was chosen for its excellent separation capabilities for peptides, allowing for efficient resolution of the target peptide from potential impurities.

Concentration: The concentration of the peptide sample that was used for HPLC analysis was not fixed, as it depended on the availability and purity of the sample obtained from the previous synthesis steps. However, we aimed to have a sample concentration that would provide a clear and distinguishable peak without overloading the column.

Sample Volume: The injection volume was 200 μL. This volume was selected to ensure a sufficiently large sample was introduced into the column for accurate detection and separation, while also considering the capacity and performance of the column.

Flow Rate: The flow rate was set at 20.00 mL/min. This relatively high flow rate was used in combination with the gradient elution program to achieve the desired separation within the specified elution time.

The gradient elution program was as follows:

From 0.00 to 1.00 min, the mobile phase consisted of 10.0% A (ACN with 0.06% TFA) and 90.0% B (water with 0.06% TFA).

From 1.00 to 8.00 min, the proportion of A changed from 10.0% to 90.0% and B from 90.0% to 10.0%.

From 8.00 to 10.00 min, it was 90.0% A and 10.0% B.

From 10.00 to 10.10 min, A decreased from 90.0% to 10.0%, and B increased from 10.0% to 90.0%.

From 10.10 to 12.00 min, it was 10.0% A and 90.0% B.

The pressure threshold was maintained between 0 and 4000 psi. The ultraviolet detector was set to monitor wavelengths at 220, 254, and 280 nm. A minimum collection threshold was set to collect the sample effluent peaks. After collection, the collected mobile-phase solution was treated by rotary evaporation under reduced pressure at 35 °C to remove acetonitrile. The remaining aqueous solution was frozen in liquid nitrogen for 20 min and then placed in a freeze-dryer for 24 h to obtain the pure product for further use.

### 5.3. The Study of the Antibacterial Properties of Melittin-Modified Peptide

*S. aereus* ATCC6538, *E. coli* ATCC25922, and *MRSA ATCC43300* were selected as the experimental strains. Bacterial suspensions were prepared for subsequent use. Four groups were established: a positive control group, a negative control group, a melittin experimental group, and a melittin-modified peptide experimental group. The concentration gradients of melittin and its modified peptide were set at 2, 4, 8, 16, 32, and 64 μg/mL. Following incubation at 37 °C for 18 h, plate counting was performed. Three sets of parallel experiments were conducted. Then, 0.5 McFarland Standards of *S. aereus* ATCC6538, *E. coli* ATCC25922, and *MRSA ATCC43300* were prepared. Melittin solutions and melittin-modified peptide solutions were subsequently prepared in concentration gradients of 51.2, 25.6, 12.8, 6.4, 3.2, and 1.6 μg/mL. These solutions were then added sequentially to a 96-well enzyme-labeled plate, with positive and negative controls established. Three sets of parallel experiments were conducted to determine and calculate the minimum inhibitory concentrations (MICs) of melittin and melittin-modified peptide against *S. aereus* and *E. coli*. We added 100 μL of solutions containing melittin and melittin-modified peptides from a 96-well enzyme-labeled plate, ensuring that their concentrations were above the minimum inhibitory concentration (MIC). We spread the solutions onto an LB nutrient agar medium and incubated at a constant temperature of 37 °C for 18 to 24 h. After incubation, we observed the growth of bacterial colonies on the plate. The concentration of melittin or melittin-modified peptide that corresponded to a colony count of ≤0.1% was defined as the minimum bactericidal concentration (MBC) against *S. aereus*, *E. coli*, and *MRSA*. We prepared suspensions of *S. aereus*, *E. coli*, and *MRSA* at a concentration of 1 × 10^8^ CFU/mL for subsequent use. Melittin and melittin-modified peptide solutions were sequentially added to wells B2 through B6 of a 96-well enzyme-labeled plate. Following this, 100 μL of the diluted bacterial suspension was sequentially added to wells B2 through B6. For the positive control wells, 50 μL of LB liquid medium and 50 μL of the diluted bacterial suspension were combined. In the negative control wells, 100 μL of 0.9% physiological saline, 100 μL of LB liquid medium, and 100 μL of sterile physiological saline were added, respectively. Three sets of parallel experiments were conducted. The samples were incubated in a constant-temperature incubator at 37 °C for 18 h, with measurements taken at 12 time points at 2 h intervals. The absorbance at 600 nm was measured to construct a bacterial growth curve. The bacterial growth curve was drawn using Graphpad Prism. The morphology and structure of the bacterial cells were examined post-treatment with melittin and its modified peptide using scanning electron microscopy

## Figures and Tables

**Table 1:** Predicted data for angle of α helix at both terminals of melittin-modified peptide.

Amino Acid	Ile	Val	Leu	Cys	Met	Phe	Trp	Tyr	Ala	Thr
α/°	98.4	98.6	97.4	98.2	96.5	97.5	98.3	96.0	97.6	97.3
Amino acid	Gly	Gln	Glu	Ser	Lys	His	Arg	Asn	Asp	Pro
α/°	96.9	95.7	95.8	96.7	95.8	96.2	95.2	95.6	96.2	96.6

**Table 2:** Comparison of the percent inhibition of different concentrations of melittin acting on three strains of bacteria.

Concentration μg/mL	2	4	8	16	32	64
*S. aereus* percent inhibition	38.16%	100%	100%	100%	100%	100%
*E. coli* percent inhibition	22.09%	30.92%	34.54%	40.16%	42.97%	37.35%
*MRSA* percent inhibition	50.34%	100%	100%	100%	100%	100%

Table note: the number of parallel tests n = 3.

**Table 3:** Comparison of antimicrobial rates of melittin-modified peptide at different concentrations among the three strains.

Concentration μg/mL	2	4	8	16	32	64
*S. aereus* percent inhibition	100%	100%	100%	100%	100%	100%
*E. coli* percent inhibition	49.40%	51.41%	55.02%	56.22%	91.16%	82.33%
*MRSA* percent inhibition	100%	100%	100%	100%	100%	100%

Table note: the number of parallel tests n = 3.

## Data Availability

The original contributions presented in this study are included in the article. Further inquiries can be directed to the corresponding authors.
